# Supplementary material for: A Critical View of the Application of the APEX Software (Aqueous Photochemistry of Environmentally-Occurring Xenobiotics) to Predict Photoreaction Kinetics in Surface Freshwaters
Source: Molecules. 2019 Dec 18;25(1):9. doi: 10.3390/molecules25010009 (PMC7017383; doi:10.3390/molecules25010009)
Supplement: Supplementary file 1 [file molecules-25-00009-s001.zip › 3.2.4_gcc-4.4.0.zip!/3.2.4_gcc-4.4.0/3.2.4_gcc-4.4.0/doc/octave/html/interpreter/Raising-Errors.html]

Raising Errors - Untitled


Next: Catching Errors,
Up: Handling Errors


---

#### 12.1.1 Raising Errors

The most common use of errors is for checking input arguments to
functions. The following example calls the `error` function if
the function `f` is called without any input arguments.

```
     function f (arg1)
       if (nargin == 0)
         error("not enough input arguments");
       endif
     endfunction
```

When the `error` function is called, it prints the given message
and returns to the Octave prompt. This means that no code following
a call to `error` will be executed.

— Built-in Function: **error** (template, ...)  
— Built-in Function: **error** (id, template, ...)  
> Format the optional arguments under the control of the template string
> template using the same rules as the `printf` family of
> functions (see Formatted Output) and print the resulting message
> on the `stderr` stream. The message is prefixed by the character
> string error: .
>
> Calling `error` also sets Octave's internal error state such that
> control will return to the top level without evaluating any more
> commands. This is useful for aborting from functions or scripts.
>
> If the error message does not end with a new line character, Octave will
> print a traceback of all the function calls leading to the error. For
> example, given the following function definitions:
>
> ```
>           function f () g (); end
>           function g () h (); end
>           function h () nargin == 1 || error ("nargin != 1"); end
> ```
>
> calling the function `f` will result in a list of messages that
> can help you to quickly locate the exact location of the error:
>
> ```
>           f ()
>           error: nargin != 1
>           error: called from:
>           error:   error at line -1, column -1
>           error:   h at line 1, column 27
>           error:   g at line 1, column 15
>           error:   f at line 1, column 15
> ```
>
> If the error message ends in a new line character, Octave will print the
> message but will not display any traceback messages as it returns
> control to the top level. For example, modifying the error message
> in the previous example to end in a new line causes Octave to only print
> a single message:
>
> ```
>           function h () nargin == 1 || error ("nargin != 1\n"); end
>           f ()
>           error: nargin != 1
> ```

Since it is common to use errors when there is something wrong with
the input to a function, Octave supports functions to simplify such code.
When the `print_usage` function is called, it reads the help text
of the function calling `print_usage`, and presents a useful error.
If the help text is written in Texinfo it is possible to present an
error message that only contains the function prototypes as described
by the `@deftypefn` parts of the help text. When the help text
isn't written in Texinfo, the error message contains the entire help
message.

Consider the following function.

```
     ## -*- texinfo -*-
     ## @deftypefn {Function File} f (@var{arg1})
     ## Function help text goes here...
     ## @end deftypefn
     function f (arg1)
       if (nargin == 0)
         print_usage ();
       endif
     endfunction
```

When it is called with no input arguments it produces the following
error.

```
     f ()
     
     -|  error: Invalid call to f.  Correct usage is:
     -|
     -|   -- Function File: f (ARG1)
     -|
     -|
     -|  Additional help for built-in functions and operators is
     -|  available in the on-line version of the manual.  Use the command
     -|  `doc <topic>' to search the manual index.
     -|
     -|  Help and information about Octave is also available on the WWW
     -|  at http://www.octave.org and via the help@octave.org
     -|  mailing list.
```

— Function File: **print\_usage** ()  
— Function File: **print\_usage** (name)  
> Print the usage message for a function. When called with no input arguments
> the `print_usage` function displays the usage message of the currently
> executing function.
>
>
>
> **See also:** help.

— Built-in Function: **usage** (msg)  
> Print the message msg, prefixed by the string usage: , and
> set Octave's internal error state such that control will return to the
> top level without evaluating any more commands. This is useful for
> aborting from functions.
>
> After `usage` is evaluated, Octave will print a traceback of all
> the function calls leading to the usage message.
>
> You should use this function for reporting problems errors that result
> from an improper call to a function, such as calling a function with an
> incorrect number of arguments, or with arguments of the wrong type. For
> example, most functions distributed with Octave begin with code like
> this
>
> ```
>           if (nargin != 2)
>             usage ("foo (a, b)");
>           endif
> ```
>
> to check for the proper number of arguments.

— Function File: **beep** ()  
> Produce a beep from the speaker (or visual bell).
>
>
>
> **See also:** puts, fputs, printf, fprintf.

— Built-in Function: val = **beep\_on\_error** ()  
— Built-in Function: old\_val = **beep\_on\_error** (new\_val)  
> Query or set the internal variable that controls whether Octave will try
> to ring the terminal bell before printing an error message.
